# Immediate Personal Supervision

**Published:** 1899-07-08

**Authors:** 


### The Hospital

A Journal of

The Medical Sciences and Hospital Administration.

Vol. XXVI.—No. 667.] Edited by Sir Henry Burdett, K.C.B., and Solomon C. Smith, M.D., M.R.C.P. [July 8, 1899.

### "Immediate Personal Supervision."

We published last week a letter from a "Medical Practitioner" with reference to the "immediate personal supervision" of unqualified assistants by medical men, and with reference also to the powers and duties of the General Medical Council in applying its requirement of such supervision to the case of midwives. Our correspondent described the practice of a lying-in charity in some unnamed locality, and expressed his belief that the medical officers of that charity were "covering" the work of unqualified persons, and were thus bringing themselves within the jurisdiction of the Council; and he also inquired whether an individual practitioner would be at liberty to "employ" midwives in a similar manner. The question turns upon two different points—first, the powers of the Council, and secondly, the broad distinction between the treatment of disease and the management of uncomplicated parturition. The only disciplinary power possessed by the Council is that of erasing from the Medical Register, and so depriving of his legal privileges as a practitioner, the name of any registered person who has been convicted of a crime or misdemeanour, or who has been found by the Council itself, after due inquiry, to have been guilty of "infamous conduct in any professional respect." The original vagueness of this phrase has been cleared off by successive judicial decisions, given on unsuccessful appeals against the judgments of the Council; and it is now settled law that if a medical man, "*in the pursuit of his profession*, has done something *with regard to it* which will be reasonably regarded as disgraceful or dishonourable by his professional brethren of good repute and competency," then it is open to the Council to say that he has been guilty of "infamous conduct in a professional respect." Now, the treatment of disease cannot be properly accomplished by any but a qualified person; and, therefore, the habitual employment of an unqualified assistant for this purpose, to visit the sick and to prescribe for them, is a fraud on the patient and his family, and may reasonably be regarded as infamous. The employment of an unqualified assistant under "immediate personal supervision," *e.g.*, to maintain extension of a fractured limb while the principal is adjusting splints, is not a fraud, and cannot reasonably be regarded as infamous, whether the unqualified assistant be a dispenser, or a pupil, or a bystander whose help is rendered available in the emergency. It cannot be brought within the accepted description of "covering," which is, "enabling an unqualified person *to practise as if he were qualified*." If we apply these considerations to midwifery, we find, in the first place, that it is an established custom in this country, dating from time immemorial, to employ a class of women called "midwives" in the management of uncomplicated parturition. Uncomplicated parturition is not a disease, the treatment of which requires medical skill and knowledge, but it is a natural process. A midwife in charge of a case of uncomplicated parturition is not discharging any medical function, nor, as things are at present, is she likely to be supposed by anyone to be a medical practitioner, although if a large number of women practitioners were to come into existence we can conceive that the case might be somewhat altered. But as things are now, there is no element of fraud in the transaction. Of late years, however, there has been a growing conviction that no women should be employed as a midwife unless she has shown her ability to distinguish complicated from uncomplicated parturition, and has been made to understand that it is her duty to obtain medical assistance as soon as the boundary line between the two has been passed. Certain bodies and persons have undertaken to give certificates of the necessary competency, more or less valid; and there is reason to believe that before long a legal standard of requirement will be established. In the meanwhile, if any medical man were to "employ" midwives—that is to say, supposing an extreme case, if he were to pay them regular wages and to send them to take charge of uncomplicated labours, assuming that he had exercised reasonable care to assure himself of their competency for this purpose, that he had ordered them to obtain his own or other medical assistance in the case of any complication occurring, and had taken care that they did so—there would be nothing in such action of which the Medical Council could take cognisance. The supposed course might be derogatory to the employer's position as a professional man, but it would be engaging in a purely commercial enterprise, in which there would be nothing "disgraceful or dishonourable," and it could not be stigmatised as "infamous," except by such a use of the word as no court of law would be likely to uphold. If the supposed medical purveyor of midwives were to be careless about their characters and qualifications, or were to permit them to remain in charge of complicated cases requiring medical skill and knowledge, then, presumably, an element of fraud or of "covering" would be introduced, and a case for the jurisdiction of the Council would be created. Without one or other of these elements, the Council would have no statutory power of interference with the natural liberty of an Englishman to conduct his business or profession in any way that may seem good to him. The Council is a body constituted by law, and subject to strict legality in all its actions. It cannot be guided by the "pious opinions" either of its own members or of the profession at large, and it cannot set up any standard of taste or any standard of ethics. Such standards, assuming them to be desirable, must be set up by the profession itself, through the instrumentality of its societies and associations.

